# Reliability and validity of the balance recovery confidence scale in persons with stroke

**DOI:** 10.3389/fnagi.2026.1813047

**Published:** 2026-05-14

**Authors:** Laksika Wangthomrong, Nithinun Chaikeeree, Wanvisa Panichaporn, Chayanee Limsuwan, Junethita Klongchant, Shawn Leng-Hsien Soh, Rumpa Boonsinsukh

**Affiliations:** 1 Faculty of Physical Therapy, Srinakharinwirot University, Bangkok, Thailand; 2 Health and Social Sciences Cluster, Singapore Institute of Technology, Singapore, Singapore

**Keywords:** fall efficacy, falls, protective strategies, psychometric properties, reactive responses

## Abstract

**Background:**

Balance recovery confidence represents a salient psychological factor influencing fall risk and functional mobility. The perceived ability to regain balance following a destabilising event may vary substantially among individuals. The Balance Recovery Confidence (BRC) Scale was developed to quantify perceived ability to regain balance across a range of destabilising situations. However, its psychometric properties have not yet been established in persons with stroke.

**Objectives:**

To translate the BRC Scale into Thai and evaluate its reliability, validity, and ability to discriminate fall history in persons with stroke.

**Methods:**

This cross-sectional study translated the BRC into Thai following established guidelines and recruited 180 individuals with subacute or chronic stroke from tertiary rehabilitation centers. Psychometric evaluations included test–retest reliability (ICC), internal consistency (Cronbach’s alpha), construct validity (Spearman’s correlations with BESTest items 14–18 and the ABC-Thai), and discriminative validity using ROC analysis with fall history as the reference.

**Results:**

The BRC-Thai demonstrated excellent test–retest reliability (ICC = 0.991) and strong correlations with BESTest items 14–18 (r = 0.766) and the ABC-Thai (r = 0.838). Internal consistency was excellent (Cronbach’s *α* = 0.985), with item–total correlations ranging from 0.825 to 0.910. Participants with a history of falls had significantly lower BRC-Thai scores than those without (*p <* 0.01). ROC analysis (AUC = 0.784) indicated good discriminative ability, with an optimal cutoff score of 95/190 (70.3% sensitivity; 78.3% specificity).

**Conclusion:**

The BRC-Thai is a reliable and valid measure of perceived reactive balance recovery in individuals with stroke.

## 1 Introduction

Stroke remains a leading neurological disorder and a major cause of long-term motor disability and reduced mobility worldwide (Langhorne et al., 2009). Although 20–66% of stroke survivors eventually regain independent ambulation, more than 80% continue to experience gait abnormalities and difficulties with daily activities such as standing, toileting, and stair negotiation (Duncan et al., 2005). These persistent impairments substantially elevate the risk of falls (Suzuki et al., 2005), which further restrict community participation and diminish quality of life. Falls represent a prevalent and persistent clinical problem across all stages of stroke recovery, with reported incidence rates ranging from 14 to 64% in the acute phase, 24–47% during inpatient rehabilitation (Baetens et al., 2011), and 23–50% in the chronic phase following discharge (Harris et al., 2005). Such high fall rates are primarily attributed to residual physical impairments, including deficits in static and dynamic balance, spasticity, motor control disturbances, and hemiparesis (Schinkel-Ivy et al., 2017). In addition, psychological factors, such as fear of falling, low fall-related self-efficacy, and diminished balance confidence during functional activities can further exacerbate fall risk (Pin et al., 2024).

Falls among individuals with stroke frequently occur during routine daily activities, particularly when tripping, slipping, or misstepping on level ground. A key contributing factor is impairment in reactive postural responses, which are automatic, adaptive mechanisms that are triggered when balance is unexpectedly disturbed to prevent a fall (Hyndman et al., 2002). Persons with stroke often exhibit substantial impairments in these reactive mechanisms, including delayed initiation or inadequate execution of protective steps, inefficient weight shifting, excessive reliance on the upper limbs for support, and limited use of the paretic limb for stepping. Such deficits compromise timely and effective balance recovery, frequently resulting in multiple unsuccessful stepping attempts and subsequent falls (Salot et al., 2016). Collectively, these impairments underscore the critical role of deficient protective stepping responses as a major determinant of elevated fall risk after stroke. The Balance Evaluation Systems Test (BESTest) provides a comprehensive assessment of postural control in persons with stroke, and items 14–18 specifically evaluate reactive postural responses (Chinsongkram et al., 2014). Scores from these items offer valuable insight into deficits of protective stepping strategies and can guide the development of targeted interventions aimed at enhancing reactive balance control.

Protective step training can be implemented through perturbation-based approaches using specialized equipment such as movable platforms or cable-release systems (Mansfield et al., 2011), or through simpler paradigms involving voluntary stepping responses (VSR) (Chayasit et al., 2022). Following such interventions, participants typically demonstrate increased step length, reduced compensatory grasping, greater use of the paretic leg for stepping, and improved postural stability (Chayasit et al., 2022; Mansfield et al., 2011). However, the effectiveness of protective step training depends on the participant’s willingness to approach the limits of stability by intentionally shifting the center of mass toward the edge of the base of support. Consequently, training outcomes are strongly influenced by an individual’s balance confidence and fall-related self-efficacy.

Fall-related self-efficacy, defined as an individual’s belief in their capability to prevent and manage falls (Soh et al., 2024a), plays a pivotal role in balance performance, postural adaptation, and response to rehabilitation. Higher levels of fall-related self-efficacy are associated with greater task participation and adaptive motor behavior, whereas reduced confidence may contribute to activity restriction, deconditioning, and elevated fall risk (Soh et al., 2024a). This construct is shaped by prior experience, motivation, and self-perception, and has been shown to correlate directly with balance control among stroke survivors (Pang and Eng, 2008). The fall-related self-efficacy continuum model conceptualizes the fall process across four stages: (Langhorne et al., 2009) the pre-fall stage, when internal or external perturbations affects an individual to perform activities steadily; (Duncan et al., 2005) the near-fall stage, characterized by imminent loss of balance requiring rapid reactive responses to prevent a fall; (Suzuki et al., 2005) the fall-landing stage, when the individual falls to a lower ground in a situation of irrecoverable balance; and (Baetens et al., 2011) the post-fall stage, encompassing on the individual’s perceived ability to get up or get help after a fall (Soh et al., 2021). The first two stages correspond to fall prevention, whereas the latter two involve fall management. Based on different fall-related self-efficacy stages, self-efficacy in each stage should be measured by different patient-reported outcome measures (PROMS). One of the most commonly used PROMS to assess balance confidence is the Activities-specific Balance Confidence (ABC) Scale (Powell and Myers, 1995). This instrument possesses strong psychometric properties and is suitable for evaluating confidence during pre-fall activities. However, because self-efficacy is context-dependent, these scales may not adequately capture confidence in reactive postural responses required during the near-fall stage. Understanding an individual’s perceived ability to recover balance following perturbations is clinically important, as this perception can be compared with actual performance to identify discrepancies and inform tailored rehabilitation planning.

To address this measurement gap, Soh et al. (2024b) developed the Balance Recovery Confidence (BRC) Scale, a 19-item patient-reported instrument designed to assess confidence in regaining balance following perturbations such as slipping, tripping, or stumbling during everyday activities. The BRC uses an 11-point Likert scale (0–10), where 0 indicates no confidence, 5 indicates moderate confidence, and 10 indicates complete confidence. The scale has demonstrated excellent test–retest reliability (ICC₃,₁ = 0.944), high internal consistency (Cronbach’s *α* = 0.975), and strong correlations with the Mini-BESTest (r ≥ 0.60). However, validation of the BRC has thus far been limited to healthy older adults without cognitive impairment, a population that differs substantially from stroke survivors who commonly present with hemiparesis, sensory deficits, and mobility limitations that may influence balance confidence.

Therefore, the present study aimed to evaluate the psychometric properties of the Balance Recovery Confidence (BRC) Scale in individuals with stroke. Specifically, test–retest reliability and internal consistency were examined, and construct validity was assessed through hypothesis testing. Convergent validity was evaluated in relation to the Activities-specific Balance Confidence (ABC) Scale, while associations with reactive balance performance were examined using items 14–18 of the Balance Evaluation Systems Test (BESTest), which assess postural responses to perturbations. Discriminative validity with respect to fall history was also investigated. As the BRC is a self-reported instrument, it was first translated and cross-culturally adapted into Thai (BRC-Thai) following established guidelines. Establishing the BRC-Thai as a valid and reliable instrument for individuals with stroke would provide clinicians with a practical tool to assess balance confidence in near-fall situations and to support fall risk screening, thereby informing more targeted and effective fall-prevention strategies.

## 2 Methodology

### 2.1 Cross-cultural translation

This study employed a cross-sectional design. With permission from the original developer, the Balance Recovery Confidence Scale (BRC) was translated and cross-culturally adapted into Thai in accordance with established guidelines (Beaton et al., 2000). The translation process involved forward translation by two bilingual translators (a physical therapist and a professional English interpreter), followed by synthesis into a single reconciled version after discrepancies were resolved. Subsequently, two independent bilingual translators, blinded to the original instrument, conducted backward translations. An expert committee comprising physical therapists with expertise in balance training, researchers, and English interpreters reviewed all versions to ensure semantic, idiomatic, experiential, and conceptual equivalence, resulting in a pre-final Thai version. This version was pre-tested with 10 individuals with stroke to assess clarity and comprehensibility, and minor revisions were made accordingly. Consequently, the final Thai version of the BRC (BRC-Thai) closely reflects the original instrument and required no substantive cultural adaptation. The BRC-Thai was then evaluated for its psychometric properties.

### 2.2 Participants

Participants with subacute (< 6 months post-onset) and chronic stroke (> 6 month post-stroke) were recruited from the rehabilitation departments of tertiary hospitals in Thailand. Sample size estimation followed COSMIN guidelines, which recommend at least 50 participants for “good” reliability testing (Mokkink et al., 2018). Based on previous studies of the original BRC, 50 participants were also deemed sufficient for assessing concurrent validity against the Balance Evaluation Systems Test (BESTest). For discriminating fall history among stroke survivors, the estimated sample size was 180 participants.

Eligibility criteria included a first cerebrovascular accident, the ability to stand independently for at least 20 min, and the ability to walk 10 meters with or without gait aids. Exclusion criteria were other neurological disorders, cognitive impairment [Montreal Cognitive Assessment (MoCA) < 18], uncorrected hearing problems, or inability to follow simple commands. All participants provided written informed consent.

### 2.3 Ethical considerations

The study protocol was approved by the Institutional Ethics Committee for Human Research, in accordance with the Declaration of Helsinki, the Belmont Report, the International Conference on Harmonization–Good Clinical Practice (ICH-GCP), the International Guidelines for Human Research, and national regulations (approval no. SWUEC-671042).

### 2.4 Procedures

Data were collected through structured patient interviews; when patients were unable to provide information, caregivers or relatives were interviewed. Demographic and clinical data included sex, age, education, affected side, stroke duration, fall history within the previous year, and relevant medical history. Additional assessments comprised spasticity (Modified Ashworth Scale; MAS), motor function (Stroke Rehabilitation Assessment of Movement–Thai version; STREAM-TH), activities of daily living (Barthel Index), cognitive function (Montreal Cognitive Assessment–Thai version; MoCA-Thai), and balance confidence (Activities-specific Balance Confidence Scale–Thai version; ABC-Thai).

Balance recovery confidence was assessed using the Balance Recovery Confidence Scale–Thai version (BRC-Thai). The primary investigator read each item aloud while presenting the corresponding scenario illustration, without providing additional explanations beyond the item statements.

The BRC-Thai was administered twice, 3 days apart under identical conditions, to evaluate test–retest reliability.

Construct validity was examined through hypothesis testing. Convergent validity was evaluated with the ABC-Thai, and associations with reactive balance performance were examined using items 14–18 of the Balance Evaluation Systems Test (BESTest). These items assess in-place postural responses and compensatory stepping following perturbations and were selected due to their conceptual alignment with the near-fall situations targeted by the BRC. Each item was performed twice, with the higher score recorded in accordance with standard BESTest administration procedures, to reflect maximal performance capacity while minimizing variability due to fatigue or inconsistent execution. Administration of each item required approximately 3–4 min, and the full BESTest (item 14–18) assessment took about 15 min. A research assistant stood on the participant’s weaker side throughout testing to ensure safety. Rest periods of 3–5 min were provided as needed to manage fatigue, during which vital signs were monitored.

The complete assessment session lasted approximately 45–60 min; for the reliability study, an additional session for the BRC-Thai conducted 3 days later required about 10 min. All assessments were performed in the morning in a quiet, dedicated room to minimize distractions.

### 2.5 Data and statistical analysis

Data were analyzed using SPSS version 26 (IBM Corp., Armonk, NY, USA). Descriptive statistics, including frequency distribution, mean, standard deviation, and percentage, were used to summarize participant characteristics and clinical variables. The Kolmogorov–Smirnov test was applied to assess data normality. As the data were not normally distributed, Spearman’s rank correlation coefficient (r) was used to examine construct validity through hypothesis testing, including convergent validity with the ABC-Thai and expected associations with reactive balance performance (BESTest items 14–18). Correlation strength was interpreted as negligible (0.00–0.10), weak (0.10–0.39), moderate (0.40–0.69), strong (0.70–0.89), or very strong (0.90–1.00) (Schober et al., 2018).

Test–retest reliability was evaluated using intraclass correlation coefficients (ICC, model 3,1) based on repeated assessments conducted 3 days apart. Reliability was interpreted as very high (ICC ≥ 0.90), high (0.75–0.90), moderate (0.50–0.74), or low (< 0.50) (Jørstad et al., 2005). Internal consistency was examined using Cronbach’s alpha coefficient, item–total correlations, and Cronbach’s alpha if item deleted. Cronbach’s alpha values were interpreted as poor (<0.60), questionable (0.60–0.69), acceptable (0.70–0.79), good (0.80–0.89), or excellent (≥0.90) (Taber, 2018).

Receiver Operating Characteristic (ROC) curve analysis was conducted using the BRC-Thai and ABC-Thai scores to determine the optimal cutoff values, area under the curve (AUC), sensitivity, and specificity, with fall history within the past year serving as the reference standard. Participants were classified as fallers or non-fallers accordingly. An AUC value of 0.80 or higher was interpreted as indicating excellent discrimination (Portney and Watkins, 2009). The optimal cutoff point was identified using the Youden index, based on the balance between sensitivity and specificity.

Likelihood ratios (LR) were used to demonstrate the post-test diagnostic accuracy, with LR^+^ values greater than 5 and LR^−^ values less than 0.2 considered clinically meaningful (Portney and Watkins, 2009). The post-test accuracy of both the BRC-Thai and ABC-Thai cutoff scores was subsequently verified against actual fall history within the same cohort.

## 3 Results

### 3.1 Test–retest reliability and construct validity

Fifty-five individuals with stroke were included in the test–retest reliability and concurrent validity analyses. Participants had a mean age of 59.33 ± 11.62 years, and 52.7% were male. The mean time since stroke onset was 111.22 ± 88.57 days; most were in the subacute stage (<6 months; 80%), with the remainder in the chronic stage (20%). Right-sided weakness was present in 50.9% of participants, and 83.6% demonstrated normal muscle tone (Modified Ashworth Scale [MAS] score = 0). Most (76.4%) were able to walk 10 m independently without gait aids, and 45.5% reported at least one fall within the previous year. The majority (70.9%) had completed primary or secondary education.

Functionally, this subgroup represented a relatively high-performing sample, with high Barthel Index scores (18.53 ± 1.67/20) and good motor function based on the STREAM (87.89 ± 13.53/100). Cognitive performance suggested mild impairment (MoCA: 23.89 ± 3.34/30). In contrast, reactive balance performance was impaired, as reflected in BESTest items 14–18 (12.62 ± 4.86/18).

The mean BRC-Thai score was 134.31 ± 48.78 at the first assessment and 135.62 ± 48.88 at the second. Test–retest reliability assessed using ICC_3,1_ was excellent (ICC = 0.991), indicating very high measurement stability.

**Figure 1:**
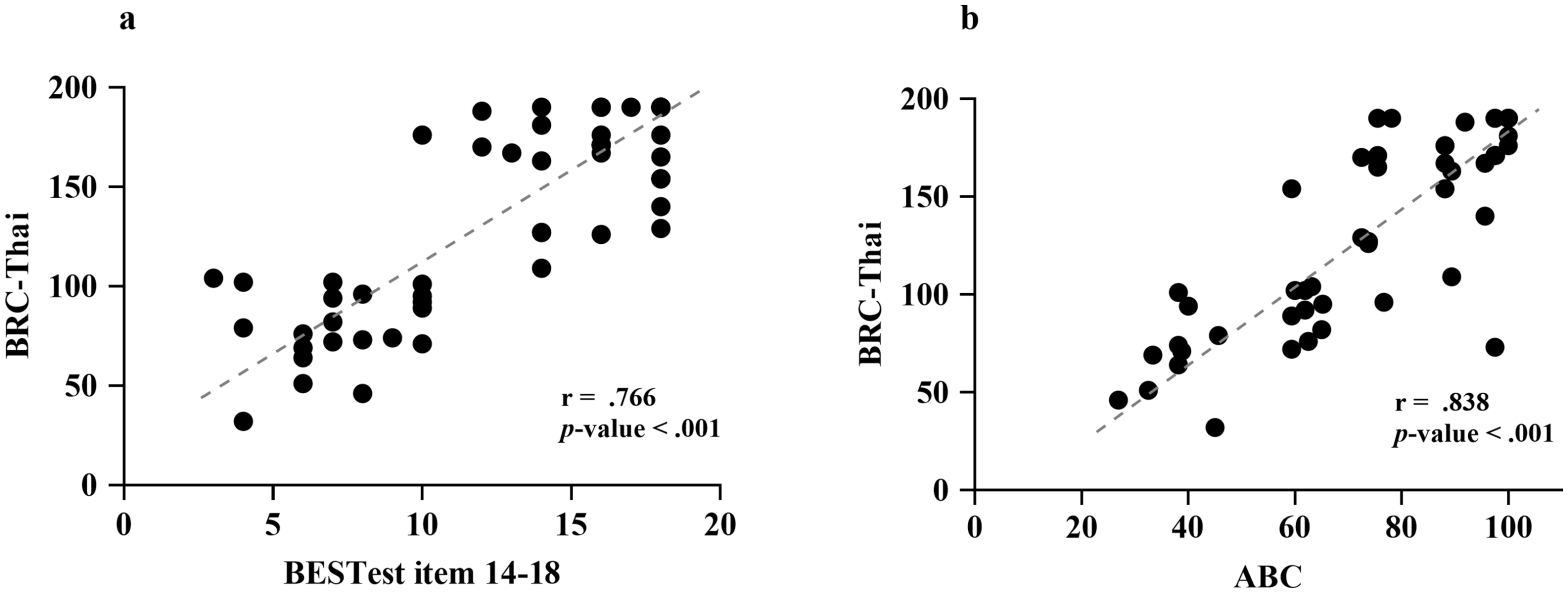
Convergent validity of the Balance Recovery Confidence Scale–Thai version (BRC-Thai). **(a)** Correlation between the BRC-Thai and the Balance Evaluation Systems Test (BESTest) items 14–18. **(b)** Correlation between the BRC-Thai and the Activities-specific Balance Confidence Scale–Thai version (ABC-Thai).

Construct validity was supported through hypothesis testing. The BRC-Thai demonstrated strong, significant positive correlations with the ABC-Thai (r = 0.838) and with reactive balance performance measured by BESTest items 14–18 (r = 0.766) (Figures 1A,B).

### 3.2 Internal consistency and discriminative validity

Internal consistency and discriminative validity were evaluated in 180 individuals with stroke, including the 55 participants who completed the test–retest and concurrent validity analyses. Participant characteristics for the full cohort and stratified by fall history are presented in Table 1. Individuals with a history of falls were significantly older, had lower educational attainment, and were more likely to use walking aids for ambulation over distances greater than 10 m. Sensory impairment and increased muscle tone (MAS ≥ 1) were also more common among participants with a fall history.

**Table 1:** Participant characteristics overall and classified by fall history.

Characteristics	Total	Faller	Non - faller
	(*N =* 180)	(*N =* 74)	(*N =* 106)
Gender^a^			
Male	81 (45)	32 (43.2)	49 (46.2)
Female	99 (55)	42 (56.8)	57 (53.8)
Age^a^	59.23 ± 9.7	62.8 ± 9.7^†^	56.7 ± 9.6
40–49 years ^b^	37 (20.6)	6 (8.1)	31 (29.2)
50–59 years ^b^	48 (26.6)	17 (23.1)	31 (29.2)
60–69 years ^b^	67 (37.2)	33 (44.6)	34 (32.2)
70–75 years ^b^	28 (15.6)	18 (24.2)	10 (9.4)
Education			
Lower than a primary	8 (4.4)	5 (6.8)	3 (2.8)
Primary	30 (16.7)	14 (18.9)	16 (15.1)
Secondary	44 (24.4)	16 (21.6)	28 (26.4)
Bachelor’s degree	95 (52.8)	37 (50)	58 (54.7)
Higher than a bachelor’s	3 (1.7)	2 (2.7)	1 (9)
Affected side^a,‡^			
Left	107 (59.4)	41 (55.4)	66 (87.7)
Right	73 (40.6)	33 (44.6)	40 (37.7)
Use a walking aid^a,‡^			
Use	80 (44.4)	34 (45.9)	45 (42.5)
No use	100 (55.6)	40 (54.1)	60 (56.6)
Sensation test ^a,‡^			
Intact	99 (55)	33 (44.6)	66 (62.3)
Impaired	81 (45)	41 (55.4)	40 (37.7)
Fall within the past year ^a, ‡^			
Yes	74 (41.1)	74 (100)	0 (0)
No	106 (58.9)	0 (0)	106 (100)
Post-stroke duration ^a^			
Less than 6 months	88 (48.9)	35 (47.3)	53 (50)
6 months to 1 year	54 (30)	23 (31.1)	31 (29.2)
More than 1 year	38 (21.1)	16 (21.6)	22 (20.8)
MAS Score ^a,†^			
0	137 (76.1)	44 (59.4)	93 (87.7)
1	34 (18.9)	21 (28.4)	13 (12.3)
2	7 (3.9)	7 (9.5)	0 (0)
3	2 (1.1)	2 (2.7)	0 (0)

MAS, Modified Ashworth Scale; ^a^Number (%); ^b^Mean ± Standard deviation. ^†^Significance between group at *p <* 0.01 (Mann–Whitney U test). ^‡^Significance between group at *p <* 0.01 (Chi-Square test).

Functional characteristics are summarized in Table 2. Participants with a history of falls had significantly lower scores on the Barthel Index, ABC scale, BRC scale, and STREAM compared with those without a fall history (*p <* 0.01). No significant between-group difference was observed in MoCA scores, with both groups demonstrating mild cognitive impairment.

**Table 2:** Functional characteristics of participants and classified by fall history.

Assessment (total score)	Total (*N =* 180)	Faller (*N =* 74)	Non-faller (*N =* 106)
ABC scale (/100)	72.1 ± 22 (11.25–100)	59.8 ± 21.6^†^ (11.3–100)	80.7 ± 17.9 (32.5–100)
BRC scale (/190)	106.1 ± 49.4 (0–190)	77.9 ± 48.4^†^ (0–190)	125.9 ± 39.8 (31–190)
Barthel ADL index (/100)	92.5 ± 10.1 (60–100)	86.6 ± 11.4^†^ (60–100)	96.6 ± 6.4 (75–100)
MoCA (/30)	22.3 ± 3 (18–30)	22.2 ± 2.97^†^ (18–28)	22.4 ± 3.1 (18–30)
STREAM (/100)	87.9 ± 14.5 (36.7–100)	80.3 ± 14.9^†^ (36.7–100)	93.2 ± 11.6 (53.3–100)

Data is presented as Mean ± SD (range). ABC, Activities-specific Balance Confidence (ABC) Scale; BRC, the Balance Recovery Confidence Scale; Barthel ADL index, Barthel Index for Activities of Daily Living; MoCA, Montreal Cognitive Assessment; STREAM, Stroke Rehabilitation Assessment of Movement. ^†^Mann–Whitney U test. Significance between group at *p <* 0.01.

Internal consistency results, including item–total correlations and Cronbach’s alpha if items were deleted, are presented in Table 3. The 19-item BRC-Thai demonstrated excellent internal consistency (Cronbach’s alpha coefficient = 0.985). Item–total correlations ranged from 0.825 to 0.916, indicating strong relationships between each item and the overall scale. Cronbach’s alpha values remained essentially unchanged when any single item was removed (0.984), suggesting that all items contribute meaningfully to the scale.

**Table 3:** Internal consistency of the BRC-Thai scale.

Items	Corrected item–total correlation	Cronbach’s *α* if item deleted
1	0.903	0.9840
2	0.858	0.9843
3	0.852	0.9844
4	0.863	0.9842
5	0.856	0.9843
6	0.871	0.9841
7	0.869	0.9841
8	0.855	0.9843
9	0.840	0.9844
10	0.825	0.9845
11	0.910	0.9839
12	0.904	0.9839
13	0.883	0.9840
14	0.878	0.9841
15	0.905	0.9838
16	0.916	0.9839
17	0.876	0.9841
18	0.907	0.9838
19	0.891	0.9840
Total	0.985	

**Figure 2:**
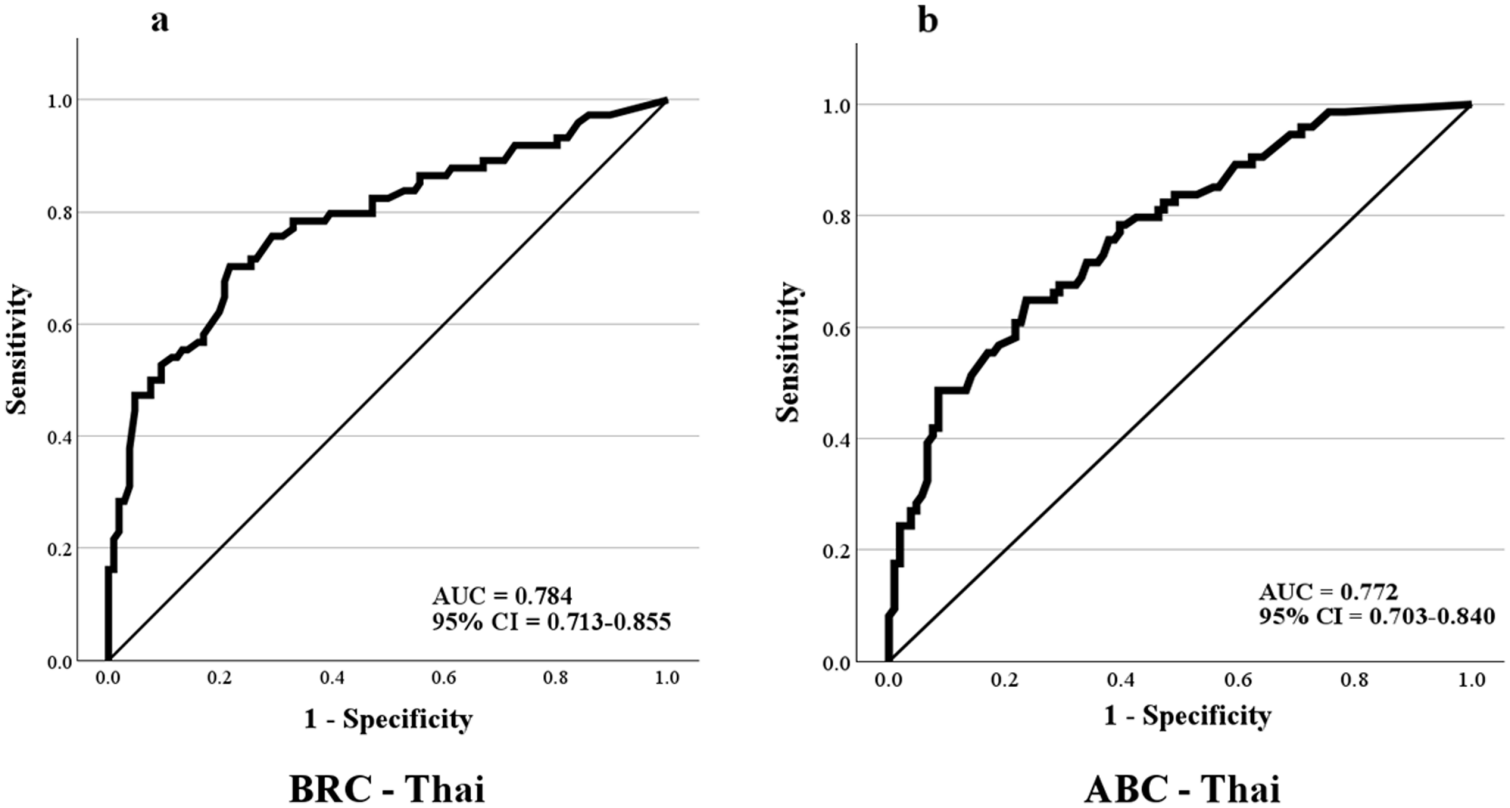
Receiver operating characteristic (ROC) analysis for discriminating fall history. **(a)** ROC curve for the BRC-Thai. **(b)** ROC curve for the ABC-Thai.

Results of the receiver operating characteristic (ROC) analysis are presented in Table 4 and Figure 2. Both the BRC-Thai and ABC-Thai demonstrated similar discriminative performance, with comparable areas under the curve (AUC). The optimal cutoff score was 95/190 for the BRC-Thai and 65/100 for the ABC-Thai. At these cutoff values, the BRC-Thai demonstrated slightly higher sensitivity, a higher positive likelihood ratio (LR+), and a lower negative likelihood ratio (LR−) than the ABC-Thai. The BRC-Thai also demonstrated greater post-test accuracy in identifying participants with a history of falls.

**Table 4:** Discriminative ability of the BRC-Thai and ABC-Thai scales to identify fall risk.

Scale	Cut point	Sensitivity	Specificity	AUC (95% CI)	Posttest accuracy	LR+	LR-
ABC	65/100	0.649	0.764	0.772 (0.703–0.840)	0.57	2.75	0.46
BRC	95/190	0.703	0.783	0.784 (0.713–0.855)	0.73	3.24	0.38

AUC, Area Under the Curve; CI, Confidence Interval; LR, Likelihood Ratio; ABC, Activities-specific Balance Confidence (ABC) Scale; BRC, the Balance Recovery Confidence Scale.

## 4 Discussion

This study is the first to comprehensively evaluate the psychometric properties of the Balance Recovery Confidence (BRC) Scale in persons with stroke. The BRC, grounded in Bandura’s self-efficacy framework, was originally developed for older adults to assess their perceived ability to recover balance following destabilizing events such as slips or trips (Soh et al., 2022). The original instrument was developed in English. For the present study, the Balance Recovery Confidence (BRC) scale was translated into Thai and culturally adapted to ensure linguistic equivalence and contextual relevance for the target population. The BRC-Thai demonstrated close conceptual and structural equivalence to the original scale, with no substantive cultural modifications required.

The BRC-Thai showed excellent measurement stability and coherence. Test–retest reliability was exceptionally high (ICC = 0.991), indicating strong temporal stability. Internal consistency was also excellent (Cronbach’s *α* = 0.985), reflecting high interrelatedness among the 19 items. Item–total correlations demonstrated that each item was strongly aligned with the underlying construct, and the minimal change in Cronbach’s alpha when any item was removed (0.984) further supported that all items contribute meaningfully to the scale. These findings closely parallel prior results in healthy older adults, where the original BRC demonstrated high test–retest reliability (ICC = 0.944) and internal consistency (Cronbach’s *α* = 0.975) (Soh et al., 2024b). Collectively, these results confirm that the BRC-Thai is a psychometrically robust and reliable tool for repeated assessment in clinical and research settings.

Construct validity was supported by a strong association between the BRC-Thai and BESTest items 14–18 (r = 0.766), which assess reactive postural responses. Although these measures represent related but distinct constructs—perceived balance recovery confidence versus observed performance—the observed relationship is consistent with Bandura’s self-efficacy framework, which posits that perceived capability is theoretically linked to actual performance. The strength of this association supports the hypothesis that individuals with greater confidence in recovering balance demonstrate better reactive balance control. The magnitude of the correlation observed in this study was higher than that reported between the original BRC and the Mini-BESTest (r = 0.51) (Soh et al., 2024b), likely reflecting the closer conceptual alignment between the BRC and BESTest items 14–18, which specifically target compensatory stepping and in-place responses during perturbations. In contrast, the Mini-BESTest encompasses broader domains of postural control, including anticipatory and dynamic balance (Franchignoni et al., 2010), which may dilute its association with near-fall self-efficacy. Importantly, this relationship should not be interpreted as evidence of construct equivalence, as discrepancies between perceived and actual balance ability may exist, particularly in populations with neurological impairments.

The BRC-Thai also showed a strong correlation with the ABC-Thai (r = 0.838), supporting convergent validity between these measures of balance confidence. While the ABC assesses confidence in performing everyday activities without falling (pre-fall situations), the BRC targets confidence in regaining stability after a perturbation (near-fall situations) (Soh et al., 2022). The stronger correlation observed in this stroke cohort compared with older adults (r = 0.54) (Soh et al., 2024b) may reflect the broader reductions in balance-related self-efficacy commonly seen in individuals with stroke, who experience persistent motor impairments affecting many aspects of postural control.

Participants with a history of falls were significantly older than non-fallers, consistent with evidence that aging contributes to deteriorations in sensory processing, neuromuscular control, and adaptability to balance disturbances (Franchignoni et al., 2010). In individuals with stroke, age-related declines are compounded by hemiparesis, abnormal tone, motor control deficits, and delayed postural reactions (Schinkel-Ivy et al., 2017). Both static balance—which relies on adequate proprioceptive, vestibular, and visual input—and dynamic balance—which requires rapid perceptual–motor integration— are therefore particularly compromised among older stroke survivors. Psychological factors, including reduced balance confidence and fear of falling, further exacerbate fall risk by limiting activity participation, restricting exposure to balance challenges, and hindering adaptive responses (Pin et al., 2024).

Consistent with this interaction, individuals with prior falls in this study exhibited poorer physical function (as reflected by lower Barthel Index and STREAM scores), greater reliance on walking aids, and reduced balance confidence. These findings align with previous work showing that greater motor impairment is associated with lower self-efficacy and higher fall risk in stroke survivors (Suzuki et al., 2005). Both the BRC-Thai and ABC-Thai demonstrated good discriminative ability for identifying individuals with a history of falls, with comparable sensitivity, specificity, and AUC values. Optimal cutoff scores were 95/190 for the BRC-Thai and 65/100 for the ABC-Thai, comparable to the 63.75/100 cutoff reported in Korean stroke survivors (Park et al., 2018). The higher post-test accuracy of the BRC-Thai (73%) may stem from its direct emphasis on common fall mechanisms such as slipping, tripping, and lateral instability (Salot et al., 2016).

Overall, this study provides evidence supporting the reliability (test–retest reliability and internal consistency), convergent validity and discriminative validity of the BRC-Thai in individuals with stroke across subacute and chronic stages. The scale is brief (5–7 min to administer), incorporates illustrated scenarios to enhance comprehension, and can be used in individuals with mild cognitive impairment. This is particularly relevant in clinical practice, as cognitive impairment is prevalent after stroke, affecting up to 60% of survivors, with approximately 30% progressing to dementia within 5 years (El Husseini et al., 2023). By capturing confidence in recovering balance during perturbations, the BRC-Thai offers clinicians a practical means of assessing psychological contributors to fall risk.

The identified cutoff scores may assist clinicians in screening for elevated fall risk and in designing interventions that address both physical and psychological aspects of balance control. Rehabilitation programs may combine reactive balance training with strategies aimed at improving confidence and reducing fear of falling, particularly in patients with low BRC scores. Item-level analysis may further help tailor treatment to specific perturbation scenarios that challenge each individual. Nonetheless, as a self-report measure, the BRC-Thai cannot replace performance-based assessments such as the BESTest or Mini-BESTest, which evaluate actual balance capacity and are less susceptible to ceiling or floor effects. A comprehensive fall risk evaluation should therefore integrate both perceived confidence and observed performance.

Several limitations should be acknowledged. Participants in this study generally exhibited relatively high functional status, with most demonstrating normal muscle tone and independent walking ability. This may partly explain the strong alignment observed between perceived and actual balance recovery ability. In contrast, prior research in older adults has reported substantial discrepancies between perceived and actual fall-arrest ability, particularly in hospitalized populations (Soh et al., 2025), which may increase fall risk when individuals overestimate their capabilities. Accordingly, future studies should examine the psychometric properties and clinical utility of the BRC-Thai in lower-functioning individuals with stroke, who may be more susceptible to such mismatches.

Fall history over the previous year was used as the reference standard for ROC analysis. Although this approach is commonly applied, retrospective fall reporting is subject to recall bias. Prospective monitoring methods, such as fall diaries, are therefore recommended in future studies to strengthen predictive validity of the BRC-Thai. In addition, this study did not account for other established fall risk factors, including muscle weakness, real-world balance performance, visual impairment, medication use, and environmental hazards. The omission of these variables may limit the comprehensiveness of fall risk assessment and the development of targeted prevention strategies. Future research should incorporate multifactorial risk profiling to enhance clinical decision-making.

## 5 Conclusion

The BRC-Thai is a reliable and valid instrument for assessing confidence in recovering balance after perturbations in individuals with stroke. Its excellent reliability, strong concurrent and convergent validity, and good discriminative ability support its use across subacute and chronic stages of recovery. The scale is brief, easy to administer, and aided by illustrated scenarios, making it feasible for routine clinical use, including among individuals with mild cognitive impairment. By capturing psychological aspects of fall risk that are not addressed by performance-based assessments, the BRC-Thai provides clinicians with valuable information for identifying individuals with reduced balance-recovery confidence and for guiding targeted, multifactorial fall-prevention strategies.

## Data Availability

The raw data supporting the conclusions of this article will be made available by the authors, without undue reservation.
